# Continuous reassortments with local chicken H9N2 virus underlie the human-infecting influenza A (H7N9) virus in the new influenza season, Guangdong, China

**DOI:** 10.1007/s13238-014-0084-6

**Published:** 2014-08-12

**Authors:** Wenbao Qi, Weifeng Shi, Wei Li, Lihong Huang, Huanan Li, Ying Wu, Jinghua Yan, Peirong Jiao, Baoli Zhu, Juncai Ma, George F. Gao, Ming Liao, Di Liu

**Affiliations:** 1 College of Veterinary Medicine, South China Agricultural University, Guangzhou, 510642 China; 2 School of Basic Medical Sciences, Taishan Medical College, Taian, 271016 China; 3 Network Information Center, Institute of Microbiology, Chinese Academy of Sciences, Beijing, 100101 China; 4 CAS Key Laboratory of Pathogenic Microbiology and Immunology, Institute of Microbiology, Chinese Academy of Sciences, Beijing, 100101 China; 5 University of Chinese Academy of Sciences, Beijing, 100049 China; 6 Beijing Institutes of Life Science, Chinese Academy of Sciences, Beijing, 100101 China; 7 Office of Director-General, (China CDC), Beijing, 102206 China

**Dear Editor**,

Since the first human infection of influenza A (H7N9) virus in Shanghai in February 2013 (Gao et al., 2013), the virus has rapidly spread out to 14 provinces of China and caused more than 139 cases of human infection with 47 deaths as of December 1, 2013 (Li et al., 2014). In 2014, 258 new cases with 99 deaths were reported (as of April 8, 2014), forming another infection peak in the new influenza season in China. Zhejiang and Guangdong Provinces have reported the majority of the cases of the new influenza season. The first re-emerging H7N9 virus was reported to have high identity to earlier ones with only five mutations in NA (Chen et al., 2013b), and implying the continuity of H7N9 infections from the previous influenza season would have featured the second H7N9 influenza virus season.

A number of studies have revealed that the H7N9 virus exhibited great diversity represented by both the surface protein-encoding genes and the internal genes during the first influenza season (Li et al., 2013; Liu et al., 2013; Shi et al., 2013; Wu et al., 2013a; Wu et al., 2013b; Wu and Gao, 2013). Particularly, the first several cases of H7N9 infection have showed both avian- and mammalian-like receptor-binding signatures and varied oseltamivir resistance (Li et al., 2013; Liu et al., 2013; Shi et al., 2013). The increased virus isolates unraveled the diversified combination of the internal genes, and thus suggested varied genotypes of viruses were co-circulating (Wu et al., 2013a). Our recent study has emphasized the genetic heterogeneity with 109 H7N9 viruses, and classified the first-season viruses into 27 genotypes (Cui et al., 2014). Surprisingly, the genetic feature was much diversified than that of H5N1 or pandemic H1N1 in their first season. The prosperity of genotypes implied the H7N9 virus had neither fixed nor fully adapted to poultry or humans, although the A/Anhui/1/2013-like viruses (genotype G0) exhibited some dominancy and were responsible for virus spread.

According to the epidemiological features of previous influenza A virus, attentions have been called to pay for the coming influenza season. It was not surprising to have human infections in the second influenza season, although great efforts, such as the closedown of live poultry markets, have been provided to prevent new infections. However, concerns were rising on whether the antigenicity and/or drug-resistance were changed, whether the virus has been fixed to either poultry or humans. For this purpose, we sequenced and collected the first re-emerging H7N9 viruses and analyzed the genetic features by means of sequence comparison, phylogenetics, genealogical reconstruction and coalescence analysis. All results indicate the re-emerging H7N9 has been experiencing continuous reassortments as in the first influenza season, however, reassorted with local chicken H9N2 virus have raised novel genotypes, which need to pay more attentions.

In this study, we are mainly focusing on the first human H7N9 infections in Guangdong and Hong Kong, where no human case was diagnosed in the first H7N9 influenza virus season. The virus isolations used in this study were from the first two human infections in Guangdong and the first, the second and the fourth human infections in Hong Kong. The first human infection in Guangdong Province (A/Guangdong/1/2013) was reported in August 2013, right between the two influenza seasons (http://www.who.int/csr/don/2013_08_11/en/index.html). The second human infection case in Guangdong (A/Guangdong/02/2013) was a three-year-old boy who had contact with live poultry (http://www.who.int/csr/don/2013_11_06/en/index.html). Hong Kong reported the first human infection case with H7N9 outside Mainland China (http://www.who.int/csr/don/2013_12_10/en/index.html), whom was a 36-year-old woman (A/Hong Kong/5942/2013) slaughtered live chicken for cooking and consuming. The patient causing the second human infection case (A/Hong Kong/734/2014) in Hong Kong was an 80-year-old man with underlying chronic illness (http://www.who.int/csr/don/2013_12_10/en/index.html). The fourth human case in Hong Kong (A/Hong Kong/2212982/2014) was a 75-year-old man with underlying illnesses who stayed with relatives living close to an LPM. All the three patients from Hong Kong had travelled to Shenzhen of Guangdong Province before the onset of illness.

**Figure 1:**
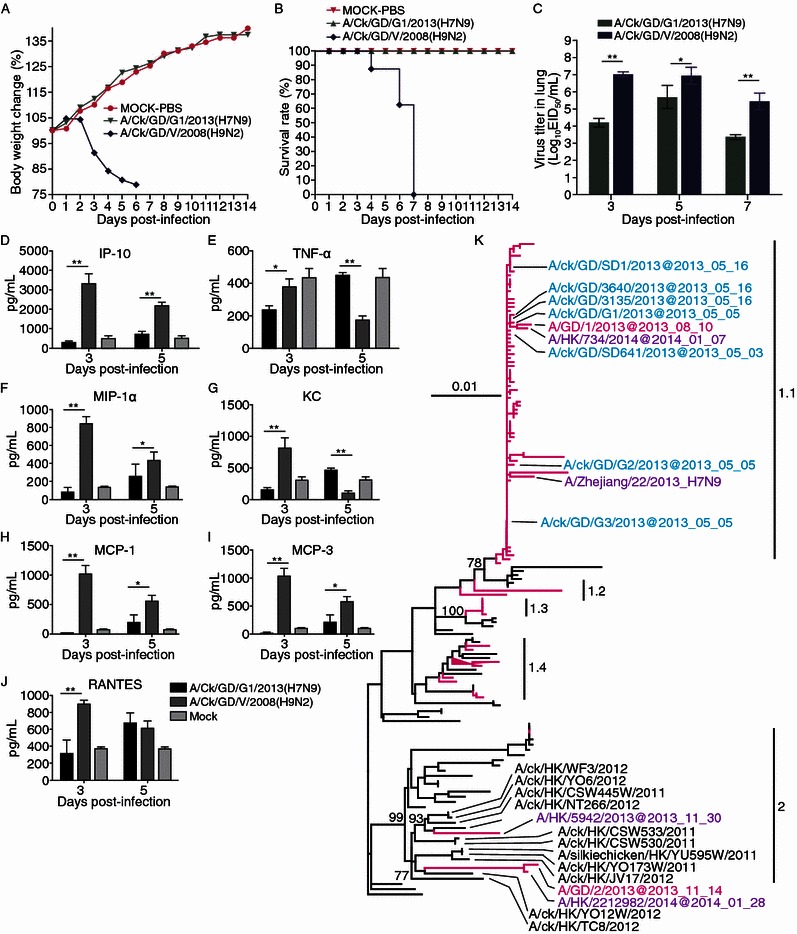
**Virus virulence, cytokine induction and phylogenetic analyses of Guangdong H7N9 virus**. (A) Weight changes of mice; (B) Mortality of mice; (C) Virus titers of the mouse lungs: five-week-old SPF BALB/c mice (seventeen mice/group) were inoculated intranasally with 10^6^ EID_50_ of each virus and three mice were euthanized on 3, 5 and 7 days post-infection and lung tissues were collected for virus titration in eggs. Groups of eight BALB/c mice were monitored daily for 14 days. Data shown are the log_10_ geometric mean EID_50_/mL ± SEM. (D–J) Cytokine IP-10, TNF-α, MIP-1α, KC, MCP-1, MCP-3 and RANTES levels from virus-infected lungs were measured at days 3, and 5 dpi by ELISA. Results from each time point are expressed as mean ± SEM of three infected mice (*n* = 3). * *P* < 0.05 and ** *P* < 0.01. (K) Maximum likelihood phylogenies of the PA gene of the new H7N9 isolates. Branches for H7N9 were in magenta and branches for H9N2 were in black. The Guangdong H7N9 viruses from poultry and the H7N9 virus of the new influenza season were also labeled. The clades of the phylogenetic tree were classified according to the branch length and bootstrap value

We isolated and sequenced H7N9 viruses from live poultry markets of Guangdong Provinces (GenBank accession: KJ395945-KJ395992). The following experiments of the virus virulence and cytokine induction in mice were also performed. As shown in Fig. 1A–C, the H7N9 isolate A/CK/GD/G1/2013 did not induce weight loss and any clinical signs, whereas the H9N2 isolate A/CK/GD/V/2008 caused severe weight loss and clinical signs including huddling, hunched posture and ruffled fur. The A/CK/GD/V/2008 virus caused 100% mortality until 7 dpi (days post-infection), whereas no mice died in the A/CK/GD/G1/2013 virus infected group. Both viruses were able to replicate in mouse lungs; however, the H9N2 virus grew to significant higher titers than the H7N9 virus. Moreover, cytokine dysregulation was associated with the pathogenicity of different subtype influence viruses, as shown by clinical, *in vivo*, and *in vitro* models (Fig. 1D–J). We analyzed 7 proinflammatory cytokines in the lungs from the mice infected by H7N9 and H9N2 viruses respectively. Lungs from the H7N9 infected mice exhibited lower levels of the proinflammatory cytokines IP-10, TNF-α, MIP-1α, KC, MCP-1, MCP-3 and RANTES at 3 dpi, but higher level of TNF-α, KC and RANTES at 5 dpi.

We noticed from the phylogenetic analyses that for the HA and NA genes, all re-emerging H7N9 viruses were clustered with the H7N9 influenza viruses from the spring outbreak, forming a single clade distinct from the other H7 and N9 viruses respectively. This implies that the re-emerging H7N9 infections in Guangdong, Zhejiang and Hong Kong were the derivatives of the earlier flu season. However, the phylogenies of the internal genes were much divergent for the re-emerging viruses, as exemplified by PA gene in Fig. 1K. Except for the PA gene of A/Guangdong/1/2013, the two human isolates from Guangdong and one from Hong Kong had all their internal genes fallen within same clades. As the patient of Hong Kong traveled to Guangdong Province (Shenzhen) before getting illness, and probably was infected by the poultry in Shenzhen, it was no surprise that those viruses were similar.

**Figure 2:**
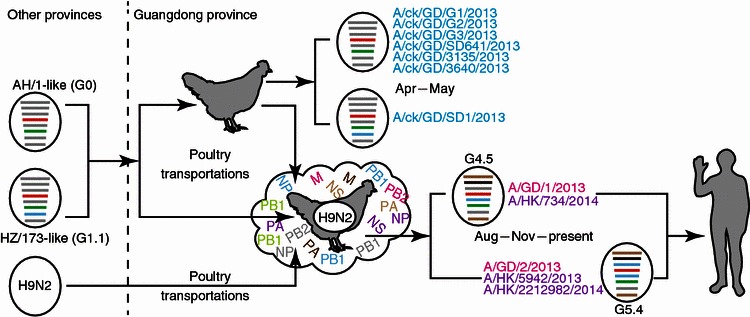
Transmission and reassortment model of Guangdong H7N9 viruses

However, the human isolates of Guangdong and Hong Kong had their internal genes very similar to those H9N2 isolates from the poultry in Hong Kong, implying that the re-emerging human isolates were reassortants of earlier H7N9 viruses and local H9N2 viruses. Despite lacking of the surveillance data of Guangdong poultry in the recent years, the historical surveillance data suggested H9N2 viruses from Guangdong and Hong Kong were closely related and those from Southern China formed a separate cluster from those isolated from Eastern and Central China (Shi et al., 2014). It should note that the H7N9 viruses were transmitted into the poultry of Guangdong Province as early as April 25 (Fig. 2). Most of the imported virus isolates (6 out of 7) were the A/Anhui/1/2013-like viruses, which were dominantly circulating in poultry and infected humans from March to June (Cui et al., 2014).

In our previous report, we established a genotype classification rule for the H7N9 virus according to the phylogenetic clades (Cui et al., 2014). And based on this classification, the six chicken isolates from Guangdong belonged to G0 and one chicken isolate belonged to G1.1. All these genotypes were found in the earlier outbreak, and the close related H9N2 internal genes to these genotypes were from Shanghai, Jiangsu and/or Zhejiang provinces (Cui et al., 2014). However, the Guangdong and Hong Kong human isolates were belonging to the genotypes that were not discovered before, as A/Guangdong/1/2013 and A/Hong Kong/734/2014 belonged to G4.5 and A/Guangdong/02/2013, A/Hong Kong/5942/2013 and A/Hong Kong/2212982/2014 belonged to G5.4. With no doubt, the novel genotypes were the results of continuous reassortment with the local H9N2 viruses. The human isolates from Guangdong and Hong Kong were most likely evolved from the poultry H7N9 viruses circulating in Guangdong. The reassortment events should occur in around three months after the H7N9 virus introduced to Guangdong according to the estimation of the most recent common ancestors.

We also compared the amino acid polymorphism of some biologically important sites of these Guangdong H7N9 viruses. Generally, these viruses did not show much amino acid variation. All the novel strains possessed 186V and nine of them possessed 226L, with one exception A/Chicken/Guangdong/SD1/2013(H7N9), holding 226Q. This implies that the novel human strains are prone to bind to mammalian receptors (Shi et al., 2013). The four human strains still possessed 292R in NA and 31N in M2, indicating they are sensitive to oseltamivir and insensitive to adamantine. As for the position 627 in PB2, all the avian isolates had E, while two human isolates had E and the remaining two human isolates possessed K. Therefore, even the virus could infect humans, some of them still could not aggregate very well in the human respiratory tract.

That the HA and NA genes were clustered together with those of previously described viruses indicated the surface protein coding genes of all of the human-infecting H7N9 viruses were originated from the same single origin. Furthermore, a number of biologically important amino acid sites in the HA and NA genes were the same as those of the spring outbreak. Therefore, the H7N9 viruses have not acquired novel genetic and antigenic features in the glycoproteins so far.

In our previous study, we analyzed a large number of the H7N9 influenza viruses available and proposed that the G0 viruses were responsible for the spread of this virus (Cui et al., 2014). Both inter-province and intra-province transmissions have led to the genesis of novel genotypes, and hence raise the genetic diversity of the H7N9 viruses. The five Guangdong chicken H7N9 viruses isolated in May belonging to G0 and G1.1 were most likely to be transported from the Yangtze River Delta following the first outbreak (Fig. 2). The study of potential transmission route based on poultry transportation would be a proof (Chen et al., 2013a). However, the human H7N9 viruses in Guangdong and Hong Kong were novel reassortants following the genetic reassortment with local poultry H9N2 viruses (Fig. 2). The phylogenetics also suggests that the H9N2 virus gene pool also consists of viruses from adjacent provinces of Guangdong of China. The estimation of the reassortment date suggests that the reassortment events would have occurred following the H7N9 introduction to Guangdong. Therefore, the H7N9 virus has continued to undergo dynamic reassortments with local H9N2 virus and caused the human infections in Guangdong and Hong Kong during the new influenza season.

It is intriguing that the dominant genotype of human isolates has changed from G0 to G4.5 and G5.4 after introduced into Guangdong, implying these new reassortants are more adaptive in both poultry and humans. The mechanism of the fitness needs further study, though Kai-Wang et al. pointed that the PA L336 M substitution would be one of the factors (Kai-Wang To et al., 2014). Poultry carrying the H9N2 virus are likely the incubators for the establishment of novel human-infecting avian influenza viruses, so that the live poultry markets and live poultry trade need to be extensively controlled (Gao, 2014; Liu et al., 2014).
